# ALDH4A1 knockdown inhibits in vitro atherosclerosis model by modulating Trim28-mediated P53 ubiquitination to suppress ferroptosis of vascular endothelial cells

**DOI:** 10.1007/s11626-025-01102-6

**Published:** 2025-08-13

**Authors:** Xiaoyong Xu, Xiaorong Xu, Wangzhuo Zhou, Wenwen Wang, Bin Lin, Xumei Huang, Shan Chen

**Affiliations:** 1 Department of Cardiology, Yongjia People’s Hospital, 283 Yongjian Road, Shangtang Town, Yongjia County, Zhejiang, 325100 China; 2 Department of Cardiology, Wenzhou Central Hospital, Zhejiang, 325000 China

**Keywords:** Atherosclerosis, Trim28, ALDH4A1, P53, Ubiquitination, Ferroptosis

## Abstract

Atherosclerosis (AS) is a primary contributor to cardiovascular disease (CVD), resulting in high mortality. Ferroptosis, triggered by lipid peroxidation, contribute to AS development. This study aimed to explore the regulatory relationships of Trim28, ALDH4A1, P53, and ferroptosis in the pathogenesis of AS. The AS cell model was constructed by treating HUVECs with oxidized low-density lipoprotein (ox-LDL). The roles of Trim28 overexpression in regulating AS development, P53 ubiquitination, and ferroptosis of vascular endothelial cells were investigated. Moreover, the interaction between Trim28 and ALDH4A1 was explored, followed by analyzing the effect of ALDH4A1 knockdown on P53 ubiquitination. Additionally, the impact of ALDH4A1 knockdown and P53 overexpression on AS development and ferroptosis of vascular endothelial cells was explored. Reduced Trim28 expression and increased ALDH4A1 and P53 expression were observed in HUVECs after treatment with ox-LDL. Overexpression of Trim28 mitigated AS development, promoted P53 ubiquitination, and suppressed ferroptosis of vascular endothelial cells. Additionally, ALDH4A1 could interact with Trim28, and ALDH4A1 knockdown enhanced P53 ubiquitination. Moreover, P53 overexpression reversed the inhibitory effects of ALDH4A1 knockdown on AS development and ferroptosis of vascular endothelial cells. Our findings indicate that Trim28, ALDH4A1, and P53 may be key regulators in AS development. Silencing of ALDH4A1 may alleviate AS development through regulating Trim28-mediated P53 ubiquitination to inhibit ferroptosis of vascular endothelial cells. These molecules may by promising therapeutic targets for AS and related CVD.

**Supplementary Information:**

The online version contains supplementary material available at 10.1007/s11626-025-01102-6.

## Introduction

Atherosclerosis (AS) is a main contributor to cardiovascular disease (CVD), which is associated with significant complications and mortality (Blagov *et al*. 2024). It is characterized by the accumulation of atheromatous plaques within the arterial walls, resulting in various clinical manifestations (Jebari-Benslaiman *et al*. 2022). Although the incidence of AS has decreased in certain countries in recent years, it remains the primary cause of death worldwide (Björkegren and Lusis 2022). Due to the profound impact on public health, it is crucial to elucidate the crucial mechanisms of AS to develop more prevention and treatment strategies.

The development of AS is influenced by various factors, with oxidative stress being a well-established initiator (Kattoor *et al*. 2017). Oxidative stress triggers endothelial cell death through multiple mechanisms, including ferroptosis (Gimbrone and García-Cardeña 2016). Ferroptosis, a form of non-apoptotic cell death, is driven by excessive oxidative damage resulting from iron overload and accumulation of lipid peroxides (Zeng and Jiang 2024). Recently, ferroptosis has been reported to play a pathological role in AS by increasing endothelial reactive oxygen species (ROS) levels and lipid peroxidation (Yang *et al.* 2021; Xu *et al*. 2024). Additionally, ferroptosis is considered as a promising target for AS treatment (Li *et al*. 2024a). However, the mechanisms underlying ferroptosis in AS development remains have not been fully elucidated at the molecular level.

Tripartite motif-containing 28 (Trim28) is involved in various biological processes, such as embryonic development, and DNA damage response (Messerschmidt *et al*. 2012; Klimczak *et al*. 2017). Trim28 can modulate endothelial cell inflammatory responses and angiogenesis (Wang *et al*. 2017). Furthermore, elevated Trim28 expression is observed in human atherosclerotic tissues, suggesting its potential role in AS (Liu *et al*. 2019). In addition, Trim28 can promote microglia ferroptosis to exacerbate neuropathic pain and neuroinflammation (Tang *et al*. 2024). Besides, accumulating evidence has revealed that Trim28 can ubiquitinate tumor protein 53 (P53) as an ubiquitin E3 ligase (Liu *et al*. 2022; Ding *et al*. 2023), and P53 plays a crucial role in mediating lipid peroxidation and ferroptosis in AS (Zhao *et al*. 2025). However, whether Trim28 contributes to AS by ubiquitinating P53 to regulate ferroptosis of vascular endothelial cells remains largely unexplored.

The human ALDH superfamily is composed of NADP + -dependent enzymes that facilitate the reversible oxidation of aldehydes to their corresponding carboxylic acids, thus safeguarding living organisms from oxidative stress (Xia *et al*. 2023). Additionally, aldehyde dehydrogenases (ALDHs) are involved in neutralizing ROS generated from the accumulation of aldehydes, effectively reducing oxidative stress in cells (Aoyama *et al*. 2013; Singh *et al*. 2013). As one member of ALDHs, aldehyde dehydrogenase 4A1 (ALDH4A1) distribution is altered during AS, and circulating ALDH4A1 is increased in mice and patients with AS, supporting the potential use of ALDH4A1 as a disease biomarker (Lorenzo *et al*. 2021). However, the role and mechanisms of ALDH4A1 in AS have not been completely clarified.

In this study, we constructed an AS cell model by exposing human umbilical vein endothelial cells (HUVECs) to oxidized low-density lipoprotein (ox-LDL). We then investigated the effects and regulatory relationships among Trim28, P53, ALDH4A1, and ferroptosis in AS in vitro. This study will provide novel insights into the molecular mechanisms underlying AS and offers potential therapeutic targets for this disease.

## Materials and methods

### Cell culture and transfection

HUVECs were obtained from Wuhan Punosai Life Technology Co., LTD (#CL-0122, Wuhan, China). HUVECs were cultured in endothelial cell medium (ECM, #SC1001-2, ScienCell, San Diego, CA) containing 10% fetal bovine serum (FBS, #16,140,071, Grand Island, NY) and 1% penicillin/streptomycin (#15,070,063, Gibco, Grand Island, NY), and incubated an atmosphere with 5% CO_2_ at 37 °C. HUVECs were cultured until they approached or reached a confluent quiescent state (HUVECs enter the quiescent phase when they reach 80–90% confluence, which was closer to the physiological environment of stable vascular endothelium). To construct an AS cell model, HUVECs were exposed to 100 μg/ml ox-LDL (#IO1300, Solarbio, Beijing, China) (Zhu *et al*. 2024; Hu *et al*. 2025). Untreated HUVECs were used as control.

The Trim28 overexpression (oe-Trim28) and p53 overexpression (oe-P53) sequences were obtained from NCBI website. The shRNA targeting ALDH4A1 (sh-ALDH4A1) sequences (sense sequence: ACTTCTACATCAACGACAA and antisense sequence: TTGTCGTTGATGTAGAAGT) were obtained in the Designer of Small Interfering RNA website. The oe-Trim28, oe-P53, sh-ALDH4A1 and their negative control (NC) were integrated into pLKO.1 lentiviral vector to construct their lentiviral vectors. To generate high-titer lentivirus, the lentiviral vectors together with pMDLg/pRRE:pVSV-G:pRSV-Rev (5:3:2) packaging plasmids were cotransfected into 293 T cells using Lipofectamine™ 2000 Transfection Reagent (#11,668,030, Invitrogen, Carlsbad, CA). The titer of oe-Trim28 lentivirus was measured to be 7 × 10^8^ TU/mL, with a multiplicity of infection (MOI) of 10. The titer of oe-P53 lentivirus was measured to be 7.4 × 10^8^ TU/mL, with a MOI of 20. The titer of sh-ALDH4A1 lentivirus was measured to be 8 × 10^8^ TU/mL, with a MOI of 10. The ox-LDL-treated HUVECs were inoculated into a 6-well plate, with 2 × 10^5^ cells per well, and cultured until the cell density reach 70–90%. Cells were then infected with oe-Trim28 lentivirals, sh-ALDH4A1 lentivirus, or/and oe-P53 lentivirus using Lipofectamine™ 2000 Transfection Reagent. After 48 h of transfection, cells were harvested.

### Cell line authentication

Authentication testing of HUVECs have been performed by Shanghai Biowing Biotechnology Co., Ltd (Shanghai, China) via STR profiling. DNA was extracted by a commercial kit from CORNING (#AP-EMN-BL-GDNA-250G, Corning, NY). The twenty STRs including Amelogenin locus were amplified by six multiplex PCR and separated on ABI 3730XL Genetic Analyzer (Thermo Fisher Scientific, Waltham, MA). The signals were then analyzed by the software GeneMapper. Mycoplasma detection of HUVECs have been performed by Shanghai Biowing Biotechnology Co., Ltd (Shanghai, China). All the experiments in this study were performed with mycoplasma-free cells.

### CCK-8 assay

A CCK-8 kit (#C0037, Beyotime, Jiangsu, China) was used for evaluating cell viability. HUVECs were seeded in a 96-well plate (2000 cells/well) and cultured at 37℃ with 5% CO_2_. After 24 hours treatment, each well was incubated with CCK-8 regent for 2 h. Absorbance at 450 nm was determined by a microplate reader (DR-3518G, Hiwell-Diatek Instruments Co., Ltd, Wuxi, China).

### Oil red O staining

Oil red O staining kit (#C01578, Beyotime, Jiangsu, China) was conducted to detect intracellular lipid accumulation. HUVECs were treated with 4% paraformaldehyde for 10 min for fixation, and stained with oil red O solution for 30 min. After staining, the plates were observed under a microscope (DMi3000 B, Leica, Wetzlar, Germany).

### Detection of total cholesterol (TC) and triglyceride (TG)

After cell supernatants collection, the TC and TG concentrations in cell supernatants were detected using the TC detection kit (#E-BC-K109-M2, Elabscience, Wuhan, China) and TG detection kit (#E-BC-K261-M1, Elabscience, Wuhan, China).

### Western blotting

Total protein was harvested using RIPA lysis buffer (#P0013B, Beyotime, Jiangsu, China). Protein concentrations were determined by a BCA kit (#P0010S, Beyotime, Jiangsu, China). The protein was isolated with a 10% SDS-PAGE and transferred onto PVDF membranes (#FFP24, Beyotime, Jiangsu, China). After sealed with non-fat milk, the membranes were incubated overnight at 37 °C with ALDH4A1 rabbit polyclonal antibody (#11,604–1-AP, 1: 1000, Proteinech, Wuhan, China), Trim28 rabbit polyclonal antibody (#PA5-27,648, 1: 1000, Thermo, Waltham, MA), recombinant anti-P53 rabbit monoclonal antibody (#ab32509, 1: 1000, Abcam, Cambridge, MA), SLC7A11/Xct rabbit polyclonal antibody (#26,864–1-AP, 1: 1000, Proteinech, Wuhan, China), GPX4 polyclonal antibody (#30,388–1-AP, 1: 1000, Proteinech, Wuhan, China), and GAPDH rabbit monoclonal antibody (#ab181602, 1: 1000, Abcam, Cambridge, MA), followed by incubation with secondary goat anti-rabbit IgG H&L (HRP) antibody (#ab6721, 1: 10,000, Abcam, Cambridge, MA) for 1 h. The proteins were visualized with an ECL chemiluminescent reagent (#P1000, Puli Lai Gene Technology Co., Ltd., Beijing, China) and quantified.

### Ubiquitination assay

The ox-LDL-treated HUVECs were first transfected with oe-Trim28, sh-ALDH4A1, or their corresponding NC. The cells were then collected for co-immunoprecipitation (co-IP) using a recombinant Anti-P53 Antibody (#ab32509, 1: 30, Abcam, Cambridge, MA), followed by immunoblotting using recombinant Anti-Ubiquitin rabbit monoclonal antibody (#ab134953, 1: 1000, Abcam, Cambridge, MA) to detect ubiquitin. The proteins were visualized with an ECL chemiluminescent reagent (#P1000, Puli Lai Gene Technology Co., Ltd., Beijing, China) and quantified.

### Co-IP assay

HUVECs were lysed on ice for 5 min using pre-cooled Lysis/Wash Buffer. The protein supernatants were collected for co-immunoprecipitation using the IP/co-IP kit (#abs9649, Absin, Shanghai, China). In brief, the rProtein A/G MagPoly Beads were incubated with anti-ALDH4A1 or anti-Trim28 antibodies for 30 min. Afterwards, cell lysate samples were added to incubate with Beads. The precipitates were incubated with IP Elution Buffer for 10 min. After incubating Neutralization Buffer, the elution fraction was collected for western blotting.

### Ferrous iron assay

HUVECs (1 × 10^5^ cells/well) were seeded in 96-well plate. An iron assay kit (#MAK025, Sigma, St. Louis, MO) was applied to determine the intracellular ferrous iron (Fe^2+^) levels in HUVECs.

### Molecular docking

The crystal structures of ALDH4A1 (3v9g) and Trim28 (2yvr) proteins were obtained from the UniProt database. Then, molecular docking was conducted using the Gramm online tool (https://gramm.compbio.ku.edu/request) (Singh *et al*. 2024). Finally, the docking results were imported into pymol software for visualization.

### Statistical analysis

All experimental data (means ± SD) were analyzed with GraphPad 8.0. Student's t tests were used to analyze differences between the two groups, and one-way analysis of variance assay for multigroup analysis. A value of P < 0.05 indicated statistically significant results.

## Results

### Detection of Trim28, ALDH4A1, and P53 expression in vascular endothelial cells treated with ox-LDL

**Figure 1.**
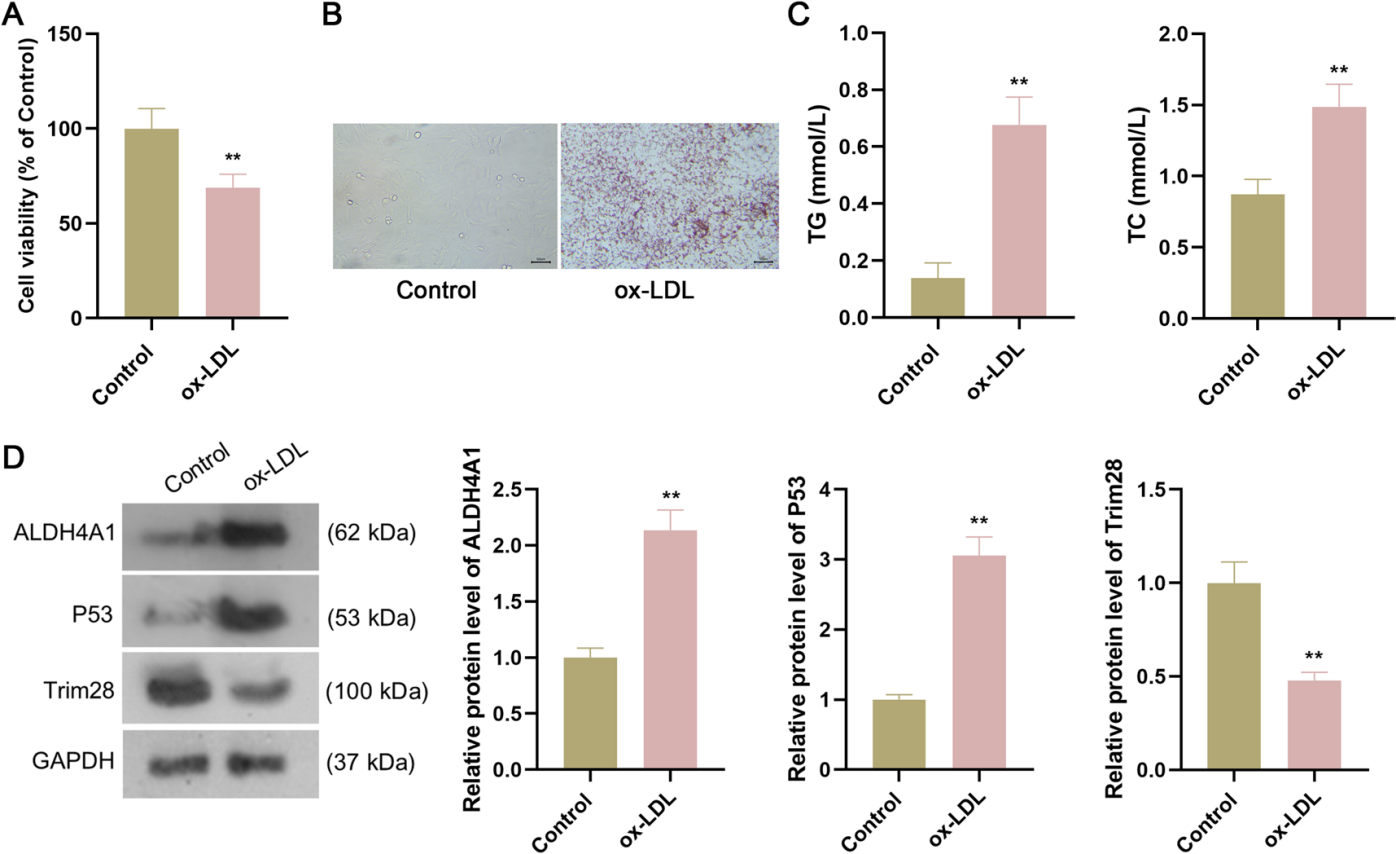
Verification of ALDH4A1, Trim28, and P53 expression in vascular endothelial cells treated with ox-LDL. (*A*): The viability of HUVECs after ox-LDL treatment. (*B*): Oil red O staining observed intracellular lipid accumulation after ox-LDL treatment. *Scale bar*: 50 µm. (*C*): TG and TC concentrations after ox-LDL treatment. (*D*): The ALDH4A1, Trim28, and P53 expression after ox-LDL treatment. Data are the means ± SD (n = 3). Student’s *t*-test was used. ** P < 0.01 compared to control group. HUVECs: human umbilical vein endothelial cells; ox-LDL: oxidized low-density lipoprotein; TC: total cholesterol; TG: total triglyceride.

To explore the expression levels of Trim28, ALDH4A1, and P53 in AS, HUVECs were treated with ox-LDL to construct the cell model of AS. Compared to control group, the viability of HUVECs was obviously decreased following ox-LDL treatment (P < 0.01, Fig. 1*A*). Oil red O staining observed intracellular lipid accumulation in the ox-LDL group, while there was no significant intracellular lipid accumulation in the control group (Fig. 1*B*). Additionally, TG and TC concentrations in cell supernatant were dramatically increased after ox-LDL treatment (all P < 0.01, Fig. 1*C*). These data indicated the AS cell model was developed. To investigate whether ALDH4A1, Trim28, and P53 were key regulators in AS development, the ALDH4A1, Trim28, and P53 expression in HUVECs treated with ox-LDL was detected. As results, Trim28 expression was markedly decreased while ALDH4A1 and P53 expression was obviously increased after ox-LDL treatment (all P < 0.01, Fig. 1*D*), demonstrating that ALDH4A1, Trim28, and P53 might be key regulators in AS development.

### Trim28 overexpression alleviated AS by mediating the ubiquitination of P53 to inhibit ferroptosis of vascular endothelial cells

**Figure 2.**
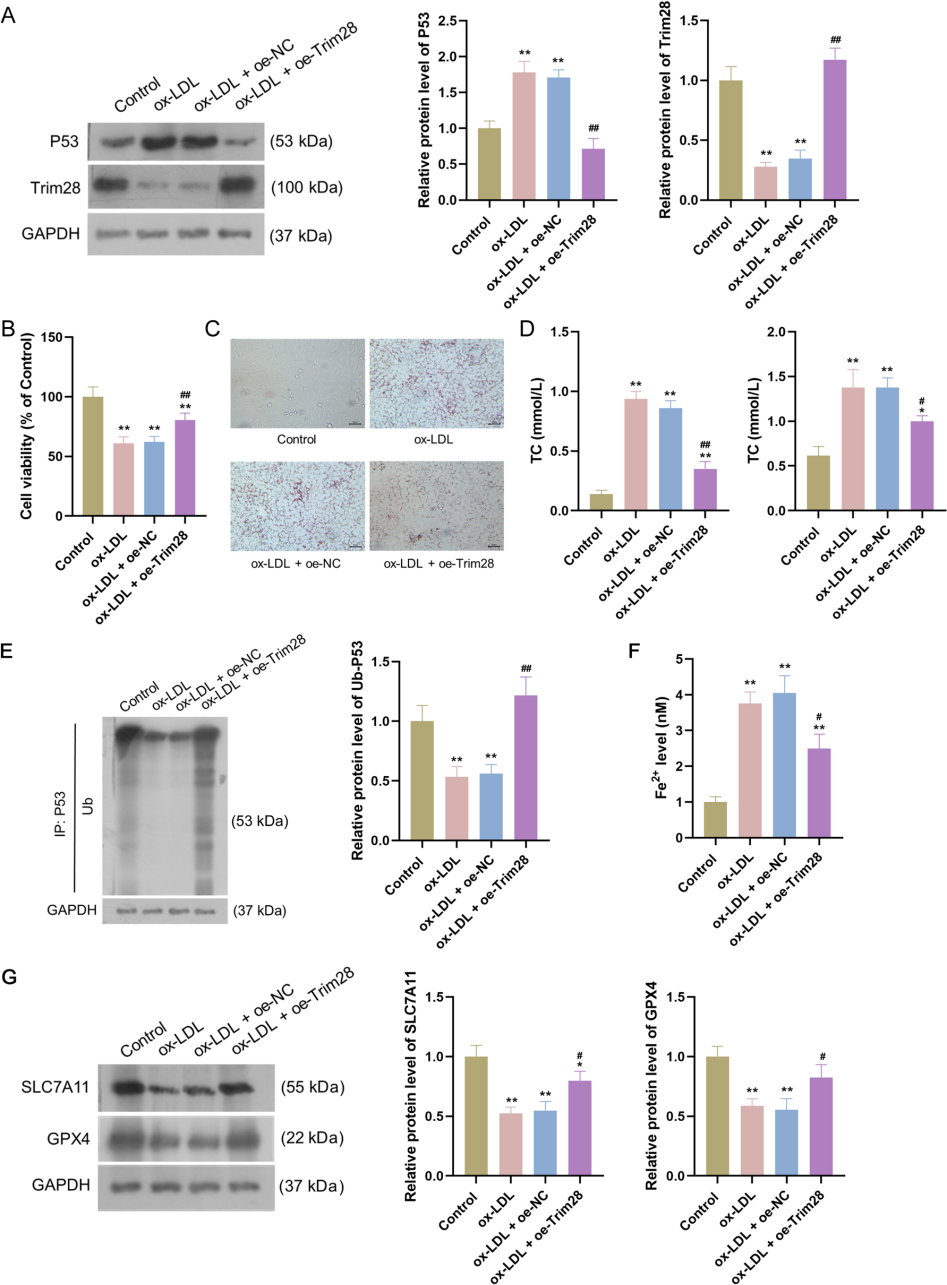
Trim28 overexpression alleviated atherosclerosis by mediating the ubiquitination of P53 to inhibit ferroptosis of vascular endothelial cells. (*A*): The protein expression of P53 and Trim28 after treatments. (*B*): The viability of HUVECs after treatments. (*C*): Oil red O staining observed intracellular lipid accumulation after treatments. *Scale bar*: 50 µm. (*D*): TG and TC concentrations after treatments. (*E*): Ubiquitination assays showed the ubiquitinated P53 levels after treatments. (*F*): The Fe^2+^ levels after treatments. (*G*): The protein expression of SLC7A11 and GPX4 after treatments. Data are the means ± SD (n = 3). One-way analysis of variance assay was used. * P < 0.05 and ** P < 0.01 compared to control group; # P < 0.05 and ## P < 0.01 compared to ox-LDL + oe-NC group. HUVECs: human umbilical vein endothelial cells; ox-LDL: oxidized low-density lipoprotein; TC: total cholesterol; TG: total triglyceride.

We further investigated the effect of Trim28 overexpression in ox-LDL-treated HUVECs. Western blotting confirmed the Trim28 was successfully overexpressed in ox-LDL-treated HUVECs following oe-Trim28 transfection (P < 0.01, Fig. 2*A*). Compared to ox-LDL + oe-NC group, cell viability was remarkably increased in the ox-LDL + oe-Trim28 group (P < 0.01, Fig. 2*B*), while intracellular lipid accumulation (Fig. 2*C*) and TG and TC concentrations (all P < 0.05, Fig. 2*D*) were dramatically reduced in the ox-LDL + oe-Trim28 group. Moreover, western blotting revealed a significant decrease in P53 expression in the ox-LDL + oe-Trim28 group relative to the ox-LDL + oe-NC group (P < 0.01, Fig. 2*A*). Ubiquitination assays also showed a significant reduction in ubiquitinated p53 levels after ox-LDL treatment, but overexpression of Trim28 notably increased ubiquitinated p53 levels (P < 0.01, Fig. 2*E*). Furthermore, cell ferroptosis was assessed under different treatment, and the expression of SLC7A11 and GPX4 (markers of ferroptosis) (Ouyang *et al*. 2022) were explored. A significant increase in Fe^2+^ levels (Fig. 2*F*) and a down-regulation of SLC7A11 and GPX4 expression (Fig. 2*G*) were observed following ox-LDL treatment (all P < 0.01), which were reversed after overexpression of Trim28 (all P < 0.05). Collectively, these results demonstrated that Trim28 overexpression could induce the ubiquitination of P53 and inhibit ferroptosis of vascular endothelial cells in AS.

### Molecular docking results

**Figure 3.**
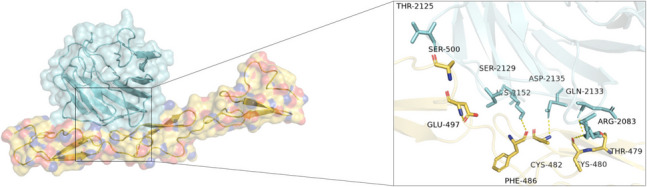
Molecular docking results of Trim28 and ALDH4A1 (input the numbered amino acids).

Molecular docking analysis revealed that the ASP-25 residue of Trim28 exhibited the highest binding energy with the SER-28 residue of ALDH4A1. Additionally, high binding energies were observed between GLY-3 of Trim28 and ALDH4A1’s ARG-20 and LEU-249 residues, along with hydrogen bond interactions between other residues (Fig. 3 and Supplementary Fig. 1). These results indicated that ALDH4A1 and Trim28 proteins had strong interaction capabilities.

### ALDH4A1 could interact with Trim28 and regulate P53 ubiquitination

**Figure 4.**
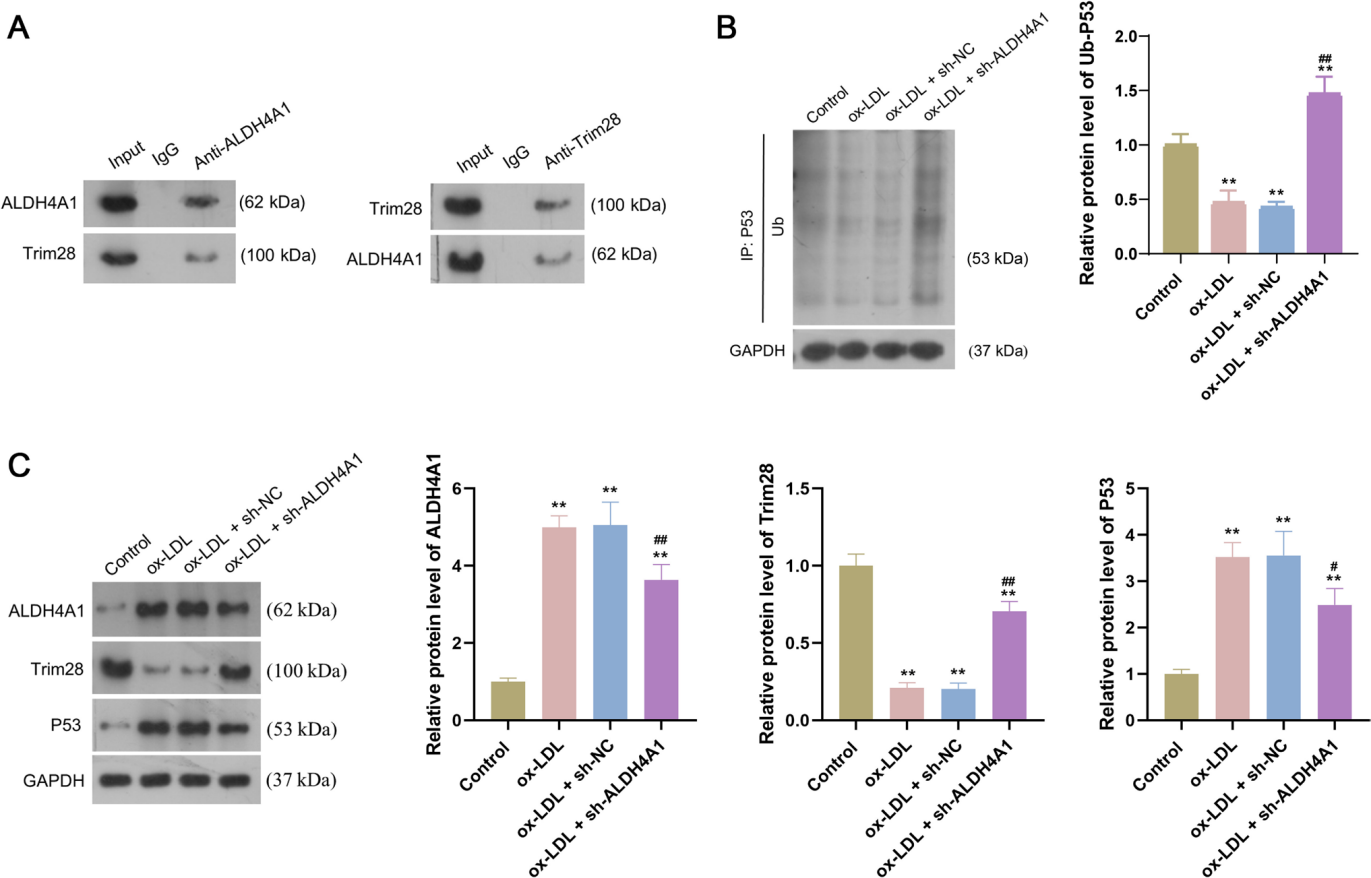
ALDH4A1 knockdown promoted Trim28-mediated P53 ubiquitination. (*A*): The co-IP assays revealed the interaction of Trim28 and ALDH4A1 in HUVECs. (*B*): Ubiquitination assay showed the ubiquitinated P53 protein level after treatments. (*C*): The protein expression of ALDH4A1, Trim28, and P53 expression after treatments. Data are the means ± SD (n = 3). One-way analysis of variance assay was used. * P < 0.05 and ** P < 0.01 compared to control group; # P < 0.05 and ## P < 0.01 compared to ox-LDL + sh-NC group. HUVECs: human umbilical vein endothelial cells; ox-LDL: oxidized low-density lipoprotein; co-IP: co-immunoprecipitation; TC: total cholesterol; TG: total triglyceride.

The co-IP assays were conducted to confirm the interaction of Trim28 and ALDH4A1 in HUVECs (Fig. 4*A*). Ubiquitination assay showed that ubiquitinated P53 protein level was significantly elevated in the ox-LDL + sh-ALDH4A1 group relative to the ox-LDL + sh-NC group (P < 0.05, Fig. 4*B*), indicating that ALDH4A1 knockdown induced the ubiquitination of P53 in ox-LDL-treated HUVECs. Moreover, western blotting assays revealed that compared to ox-LDL + sh-NC group, knockdown of ALDH4A1 decreased ALDH4A1 and P53 expression and increased Trim28 expression in the ox-LDL + sh-ALDH4A1 group (all P < 0.05, Fig. 4*C*). Taken together, ALDH4A1 knockdown could induce Trim28-mediated P53 ubiquitination.

### Overexpression of P53 attenuated the inhibitory effect of ALDH4A1 knockdown on AS development and ferroptosis of vascular endothelial cells

**Figure 5.**
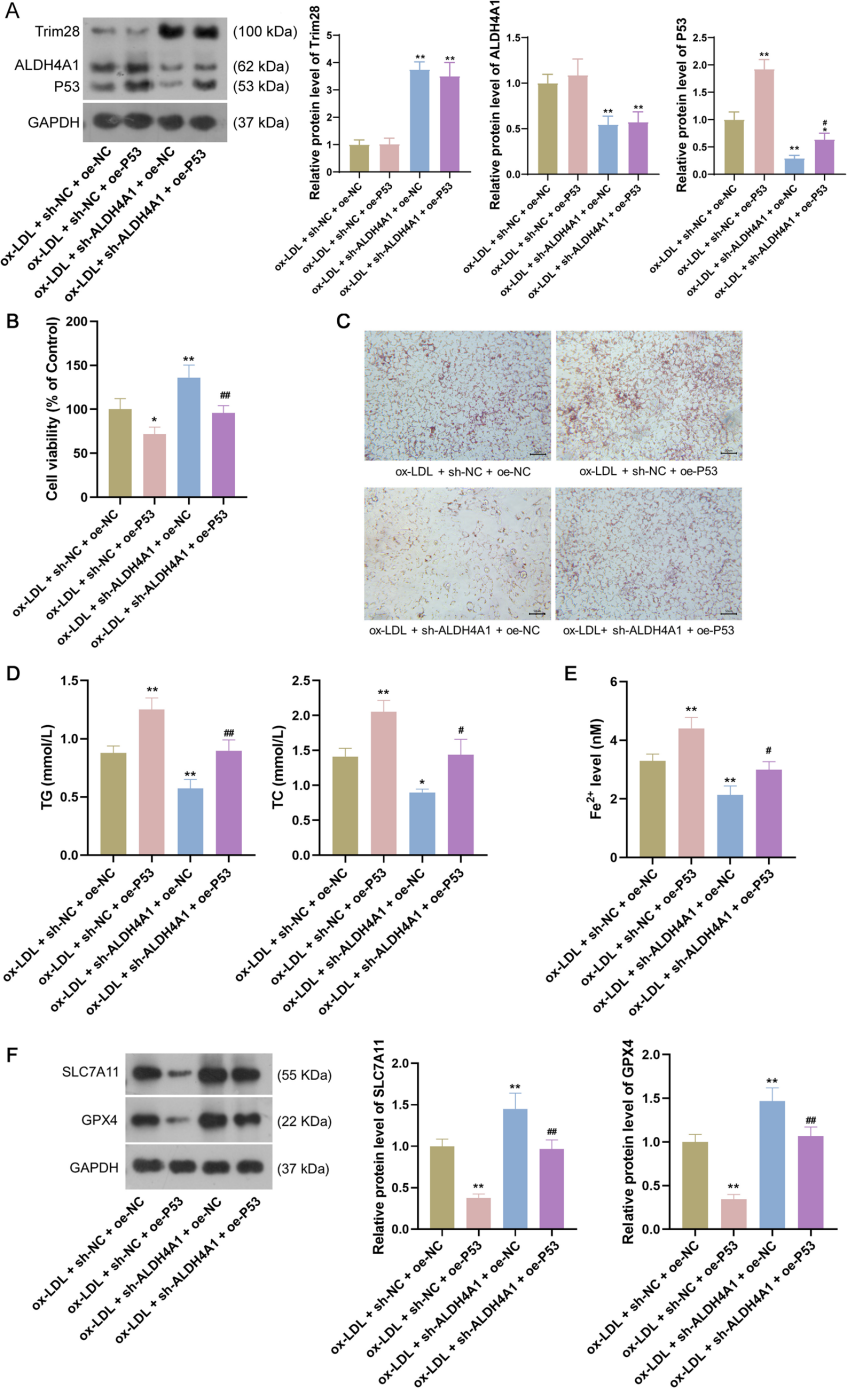
Overexpression of P53 attenuated the inhibitory effect of ALDH4A1 knockdown on AS by promoting ferroptosis of vascular endothelial cells. (*A*): The protein expression of Trim28, ALDH4A1 and P53 expression after treatments. (*B*): The viability of HUVECs after treatments. (*C*): Oil red O staining observed intracellular lipid accumulation after treatments. *Scale bar*: 50 µm. (*D*): TG and TC concentrations after treatments. (*E*): The Fe^2+^ levels after treatments. (*F*): The protein expression of SLC7A11 and GPX4 after treatments. Data are the means ± SD (*n* = 3). One-way analysis of variance assay was used. * P < 0.05 and ** P < 0.01 compared to ox-LDL group; # P < 0.05 and ## P < 0.01 compared to ox-LDL + sh-ALDH4A1 + oe-NC group. HUVECs: human umbilical vein endothelial cells; ox-LDL: oxidized low-density lipoprotein; TC: total cholesterol; TG: total triglyceride.

The effects of ALDH4A1 knockdown on P53 expression, AS development, and ferroptosis of vascular endothelial cells were detected. Compared to ox-LDL + sh-NC + oe-NC group, decreased P53 expression (P < 0.01, Fig. 5*A*), elevated cell viability (P < 0.01, Fig. 5*B*), reduced intracellular lipid accumulation (Fig. 5*C*) and TG and TC concentrations (all P < 0.05, Fig. 5*D*) were observed in the ox-LDL + sh-ALDH4A1 + oe-NC group, demonstrating that ALDH4A1 knockdown inhibited P53 expression and AS development. Moreover, the ox-LDL + sh-ALDH4A1 + oe-NC group exhibited lower Fe^2+^ levels (P < 0.05, Fig. 5*E*) and higher SLC7A11 and GPX4 expression (all P < 0.01, Fig. 5*F*) than ox-LDL + sh-NC + oe-NC group, revealing that ALDH4A1 knockdown suppressed ferroptosis of vascular endothelial cells.

To determine whether the role of ALDH4A1 in AS was mediated by P53, we overexpressed P53 and investigated the combined effects of ALDH4A1 knockdown and P53 overexpression in ox-LDL-treated HUVECs. Western blotting showed that P53 was successfully overexpressed following transfection with oe-P53 (P < 0.01), but P53 overexpression did not significantly affect ALDH4A1 expression (Fig. 5*A*). Compared to ox-LDL + sh-ALDH4A1 + oe-NC group, P53 expression was remarkably increased in the ox-LDL + sh-ALDH4A1 + oe-P53 group (P < 0.05, Fig. 5*A*), indicating that P53 was successfully overexpressed after transfected with oe-P53. Additionally, compared to ox-LDL + sh-ALDH4A1 + oe-NC group, cell viability (P < 0.01, Fig. 5*B*) was remarkably reduced while the intracellular lipid accumulation (Fig. 5*C*) and TG and TC concentrations (all P < 0.05, Fig. 5*D*) were notably increased in the ox-LDL + sh-ALDH4A1 + oe-P53 group, indicating that P53 overexpression might mitigate the inhibitory effect of ALDH4A1 knockdown on AS development. Furthermore, relative to ox-LDL + sh-ALDH4A1 + oe-NC group, the ox-LDL + sh-ALDH4A1 + oe-P53 group exhibited higher Fe^2+^ levels (P < 0.05, Fig. 5*E*) and lower SLC7A11 and GPX4 expression (all P < 0.01, Fig. 5*F*), indicating that P53 overexpression attenuated the inhibitory effect of ALDH4A1 knockdown on ferroptosis of vascular endothelial cells.

## Discussion

AS is a chronic disorder that is connected to oxidative stress imbalance and lipid metabolism abnormalities. Ferroptosis, triggered by lipid peroxidation, has been shown to contribute to inflammation and oxidative stress, both of which play a key role in AS development (Wang *et al*. 2021). Therefore, elucidating the key mechanisms mediating ferroptosis in AS could facilitate potential therapeutic approaches for AS and related diseases. In this study, we found the aberrant expression of Trim28, ALDH4A1, and P53 in HUVECs following ox-LDL treatment. Inhibition of ALDH4A1 attenuated AS development through modulating Trim28-mediated P53 ubiquitination to suppress cell ferroptosis. These findings suggest that targeting Trim28, P53, and ALDH4A1 is of great significance for preventing endothelial cell ferroptosis and mitigating AS progression.

Accumulating evidence indicates that ubiquitination plays a crucial role in various processes like lipid metabolism, endothelial and smooth muscle cell function, and vascular inflammation in the pathogenesis of AS (Zhang *et al*. 2018). E3 ubiquitin ligases are essential regulators in numerous diseases, including AS (Zhou *et al*. 2021). As a key E3 ubiquitin ligase, Trim28 functions a central player in endothelial inflammatory responses and angiogenesis in endothelial cells (Wang et al. 2017). Additionally, Trim28 enhances the epigenetic activation of T helper 17 (Th17) cells. Deficiency of Trim28 can impair Th17 differentiation and alleviate inflammatory diseases (Jiang *et al*. 2018). Th17 differentiation plays a key role in AS (Lin *et al*. 2013). Our study revealed that Trim28 was down-regulated in ox-LDL-treated HUVECs, and Trim28 overexpression alleviated AS development. These data indicate that Trim28 may be a key regulator in AS development.

Consistent with previous findings (Jin *et al*. 2021; Liu *et al*. 2022; Ding *et al*. 2023), we also found that Trim28 overexpression induced P53 ubiquitination. P53 is a key transcription factor that responds to cellular stress, and plays a fundamental role in regulating antioxidant activity (Cenini *et al*. 2008). P53 participates in antioxidant defenses through multiple mechanisms, such as directly activating the transcription of antioxidant genes, initiating apoptosis, and promoting the production of enzymes that neutralize ROS (Lee *et al*. 2022; Zeng *et al.* 2023; Zhu *et al*. 2023). Moreover, P53 is involved in numerous metabolic processes, like lipid metabolism, glycolysis, and protein degradation. These processes are essential for cellular functions, thereby contribute to AS development and progression (Lu *et al*. 2023). In addition, P53 could affect ferroptosis via regulating GPX4 expression and modulating its activity (Huang *et al*. 2023; Zhu *et al*. 2023). Furthermore, P53 is involved in ferroptosis via modulating downstream targets, like SLC7A11. These targets help facilitate the import of cysteine into cells, promoting the production of glutathione (GSH) and thereby protecting cells from ferroptosis (Qian *et al*. 2022). In our study, P53 expression was remarkably increased in ox-LDL-treated HUVECs, confirming the potential role of P53 in AS development. Notably, we observed that Trim28 overexpression induced P53 ubiquitination and inhibited ferroptosis of vascular endothelial cells. Given the key role of P53 in cell ferroptosis, it can be speculated that Trim28 overexpression may alleviate AS development via ubiquitinating P53 to inhibit ferroptosis of vascular endothelial cells.

Furthermore, this study revealed that Trim28 could interact with ALDH4A1. As one member of ALDHs, elevated circulating ALDH4A1 levels have been observed in patients with AS (Lorenzo *et al*. 2021), suggesting that ALDH4A1 might act as a potential biomarker for AS diagnosis. In circulation and atherosclerotic plaque tissue, the presence of auto-antigen ALDH4A1 may cause an immune response in the spleen, resulting in the production of specific antibodies by B lymphocytes. Compared to plaque rupture, patients with plaque erosion exhibited higher ALDH4A1 expression in plasma and coronary thrombi, pointing to its potential contribution to AS and plaque erosion (Li *et al*. 2024b). Herein, ALDH4A1 was elevated in HUVECs after ox-LDL exposure. Knockdown of ALDH4A1 increased P53 ubiquitination, and P53 overexpression mitigated the effects of ALDH4A1 knockdown on AS and endothelial ferroptosis. These findings demonstrated that ALDH4A1 plays a crucial protective role in AS through modulation of Trim28-mediated P53 ubiquitination.

This study was the first to reveal the interaction between Trim28 and ALDH4A1 as well as demonstrate their regulatory effects on P53 ubiquitination and ferroptosis in AS, providing new insights into promising mechanisms underlying AS. However, this research also has several limitations. First, the Trim28, P53, and ALDH4A1 expression was not validated in clinical samples. The clinical applicability of these markers for the AS diagnosis should be further validated using additional clinical cohorts. Second, in vivo assays were not performed to clarify the regulatory relationships among Trim28, P53, ALDH4A1, and ferroptosis. More research should not conduct to verify these findings. In addition, the association between P53 and ferroptosis should be further deeply explored.

**Figure 6.**
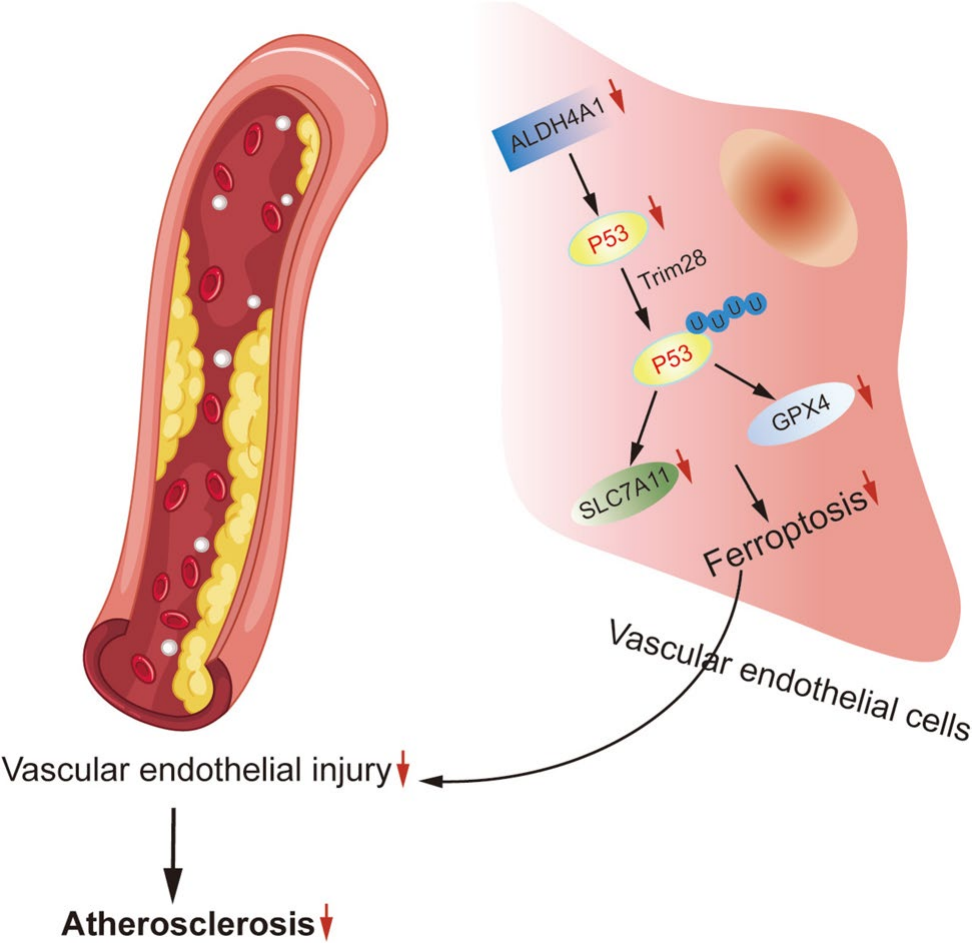
Mechanism diagram.

In conclusion, our findings revealed that Trim28, ALDH4A1 and P53 are implicated in the pathogenesis of AS. Trim28 overexpression may alleviate AS development via ubiquitinating P53 to inhibit ferroptosis of vascular endothelial cells. Additionally, ALDH4A1 knockdown may inhibit ferroptosis in AS through modulation of Trim28-mediated P53 ubiquitination (Fig. 6). These findings open up new avenues for targeted therapies aimed at preventing or alleviating the vascular complications associated with AS.

## Supplementary Information

Below is the link to the electronic supplementary material.

Supplementary Figure 1 Molecular docking results of Trim28 and ALDH4A1 (PNG 2.37 MB)

High Resolution Image (TIF 3.58 MB)

## Data Availability

All data are included in the manuscript. Upon requests to the corresponding author.
