# Supplementary figures and images for: ALDH4A1 knockdown inhibits in vitro atherosclerosis model by modulating Trim28-mediated P53 ubiquitination to suppress ferroptosis of vascular endothelial cells
Source: In Vitro Cell Dev Biol Anim. 2025 Aug 13;61(9):1110–9. doi: 10.1007/s11626-025-01102-6 (PMC12628478; doi:10.1007/s11626-025-01102-6)

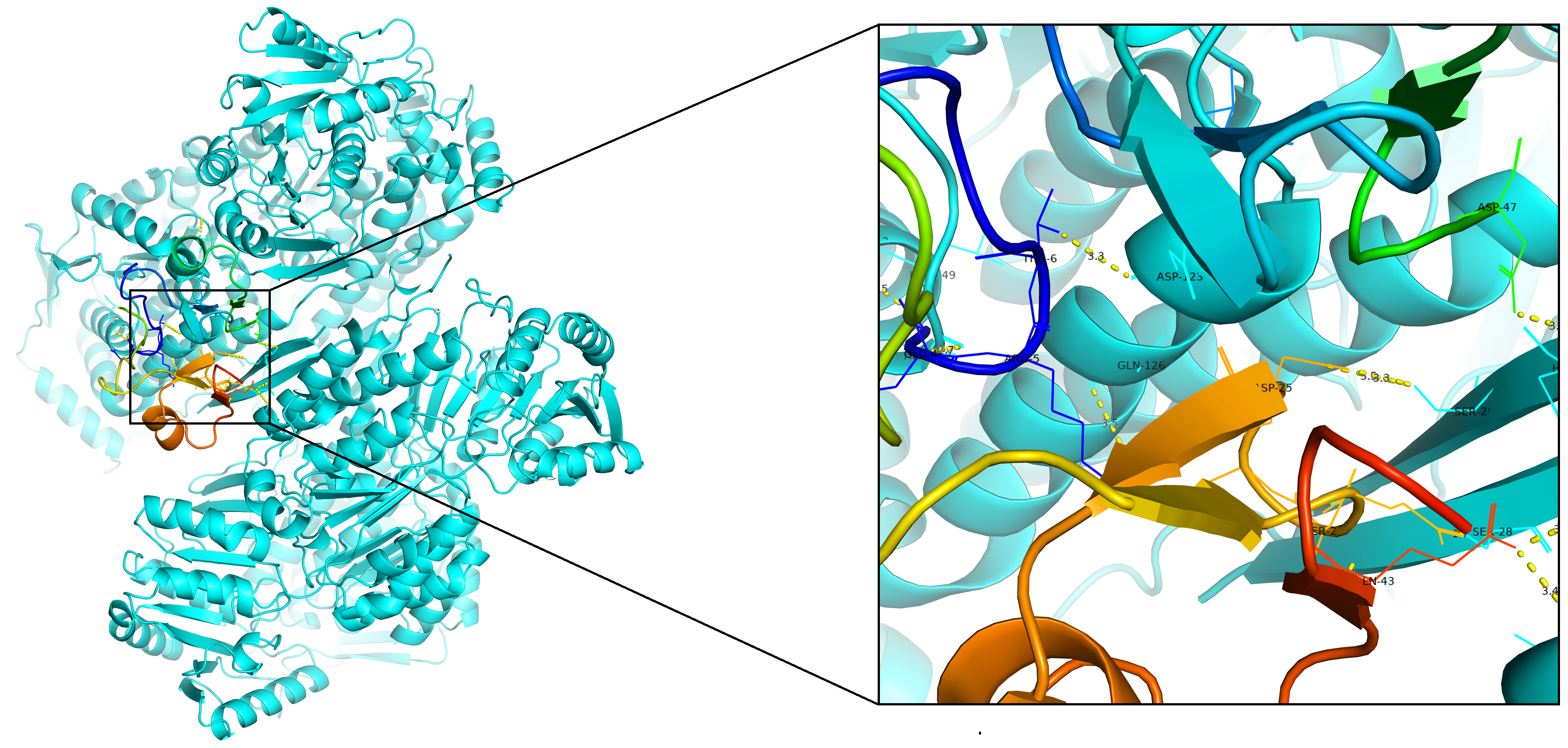

Supplement: Supplementary file 1 — Molecular docking results of Trim28 and ALDH4A1 (PNG 2.37 MB) [file 11626_2025_1102_Fig7_ESM.png]

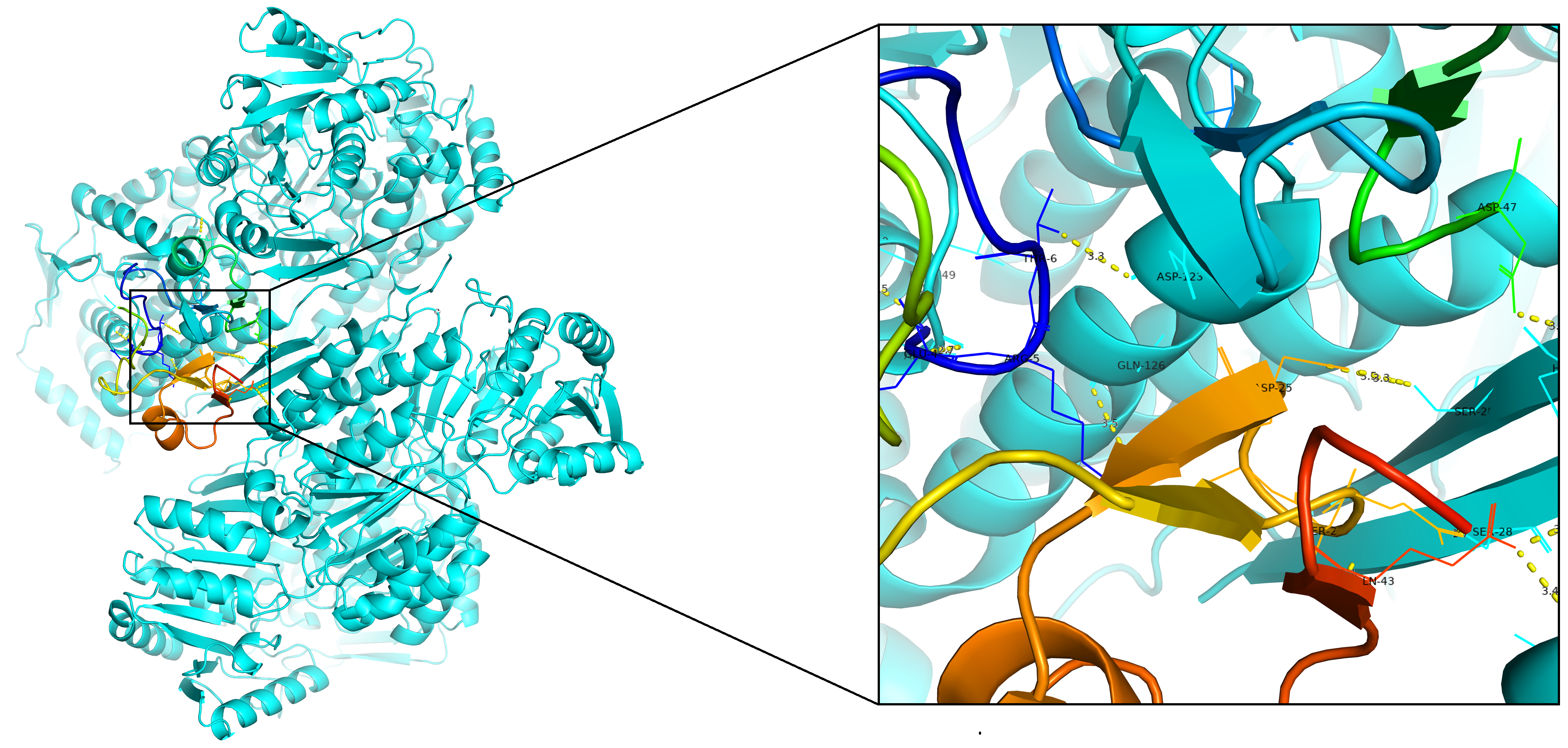

Supplement: Supplementary file 2 — High Resolution Image (TIF 3.58 MB) [file 11626_2025_1102_MOESM1_ESM.tif]
